# Supplementary material for: Single cell genome sequencing of laboratory mouse microbiota improves taxonomic and functional resolution of this model microbial community
Source: PLoS One. 2022 Apr 13;17(4):e0261795. doi: 10.1371/journal.pone.0261795 (PMC9007364; doi:10.1371/journal.pone.0261795)
Supplement: S1 Fig — Multiple metrics are improved when comparing high coverage versus low coverage single cell sequencing data. Facets show the individual metrics assessed: assembly completeness as determined by CheckM, percentage of reads filtered out as contaminants, total length of the genome assembly in base pairs (bp), maximum contig length (in bp), total number of reads generated. Numbers over each boxplot represent p-values of paired Wilcoxon rank sum tests. (PDF) [file pone.0261795.s003.pdf]

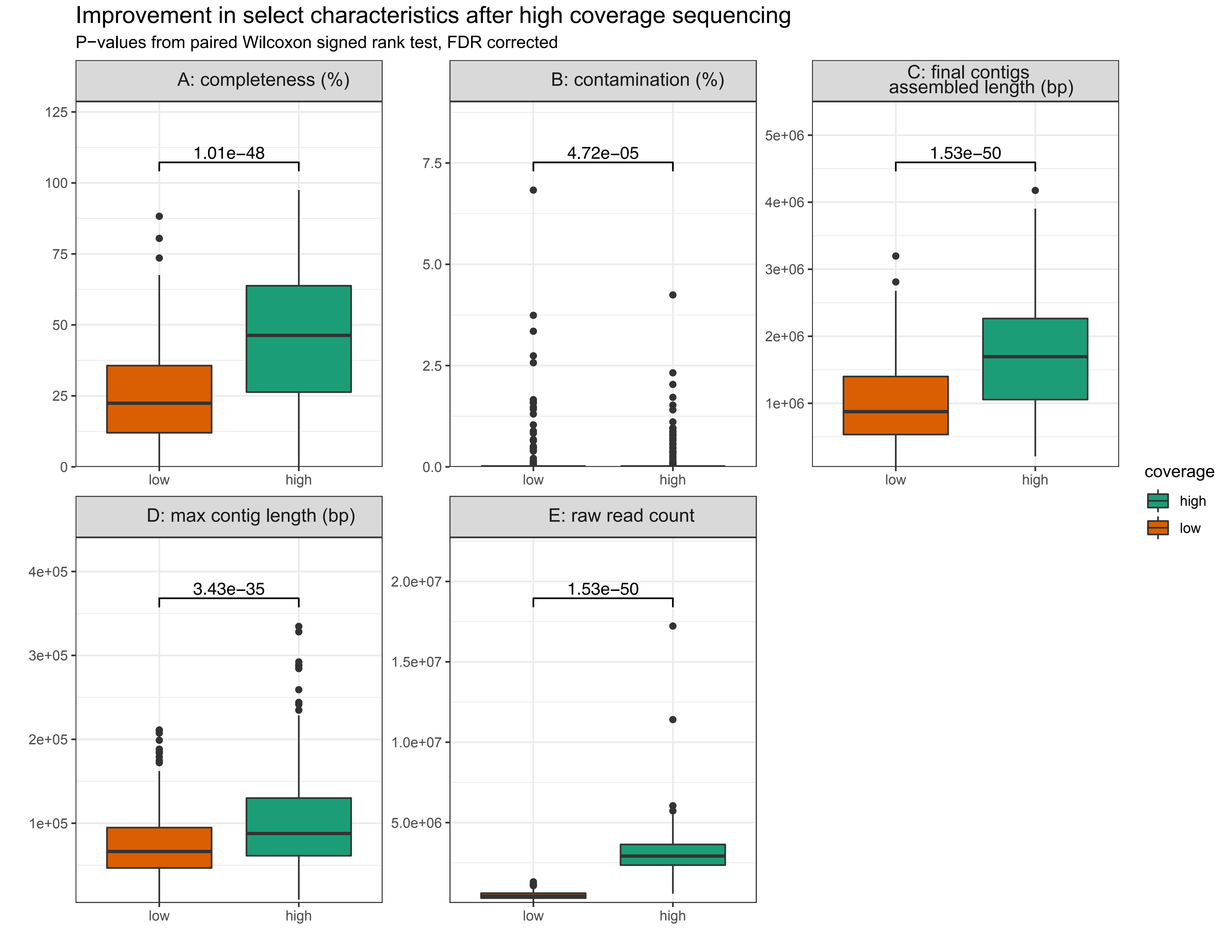
Improvement in select characteristics after high coverage sequencing
P−values from paired Wilcoxon signed rank test, FDR corrected
A: completeness (%)
1.01e−48
B: contamination (%)
4.72e−05
C: final contigs assembled length (bp)
1.53e−50
D: max contig length (bp)
3.43e−35
E: raw read count
1.53e−50
low
high
coverage
high
low
