# Supplementary material for: Single cell genome sequencing of laboratory mouse microbiota improves taxonomic and functional resolution of this model microbial community
Source: PLoS One. 2022 Apr 13;17(4):e0261795. doi: 10.1371/journal.pone.0261795 (PMC9007364; doi:10.1371/journal.pone.0261795)
Supplement: S2 Fig — Ridgeline plots representing distributions of 2 metagenomic classifier performance metrics when using sourmash—total number of kmer hashes assigned and number of species with more than 5 kmer hashes (an approximation for prevalence). Ridgeline plots are a form of multi-distribution density plot that vertically separate the individual distributions to improve clarity in cases of overlap, necessitating the removal of the traditional y axis label (“density”). This renders the densities not directly comparable between individual ridge lines, but aids in assessment of differences in skew, bimodality, and potential pronounced shift of distribution peaks.The plots are faceted by test metagenomic dataset, and each line within the facet reflects one of the three reference database options—default set of genomes available in GTDB release 86 (labeled “gtdb_ref”), a custom database with single-cell genomes only (labeled “scg_ref”), and a combined database with the GTDB r86 and single-cell genomes (labeled “combined_ref”). (PDF) [file pone.0261795.s004.pdf]

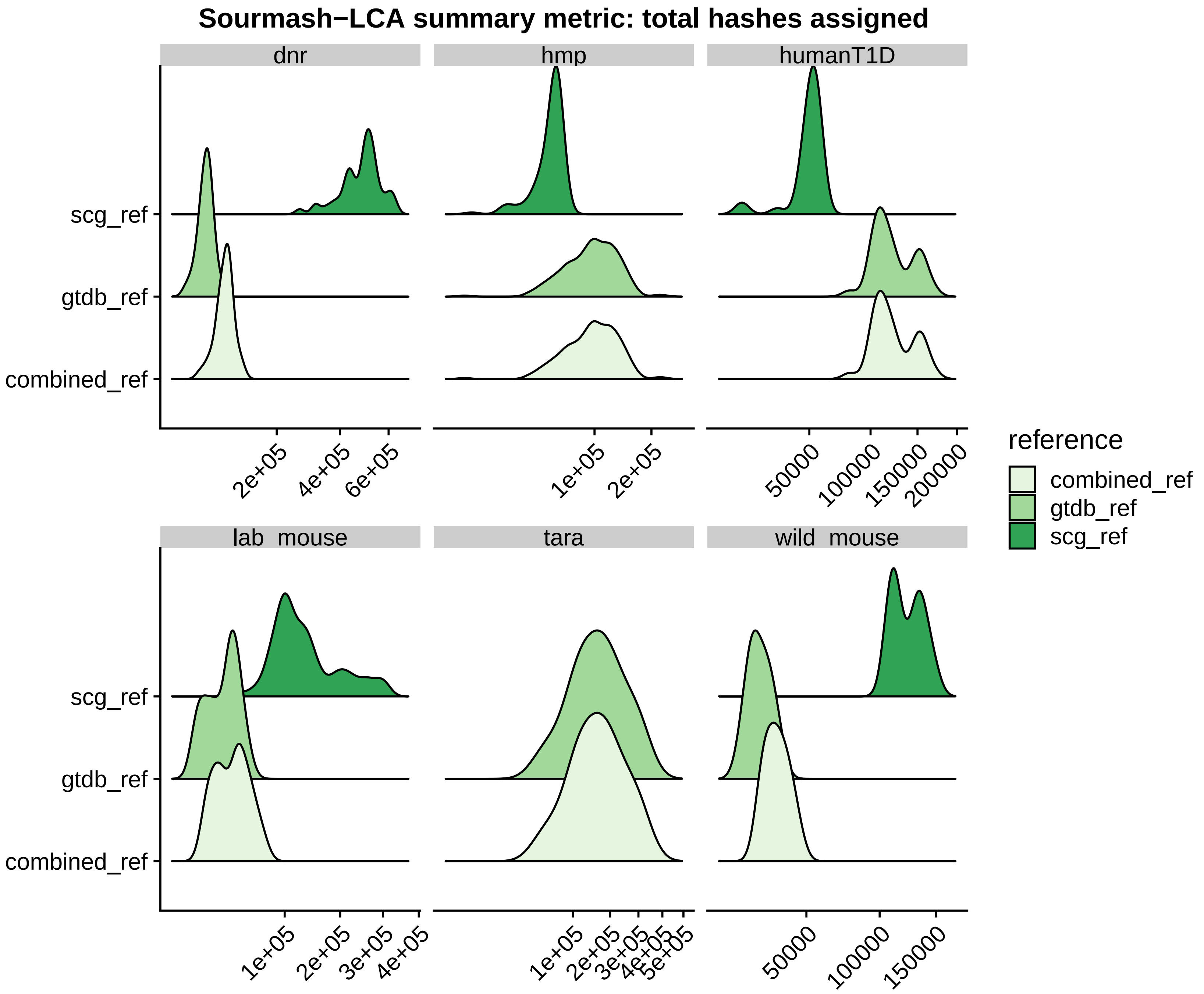
Sourmash−LCA summary metric: total hashes assigned
dnr
hmp
humanT1D
lab_mouse
tara
wild_mouse
scg_ref
gtdb_ref
combined_ref
2e+05
4e+05
6e+05
1e+05
2e+05
50000
100000
150000
200000
1e+05
2e+05
3e+05
4e+05
1e+05
2e+05
3e+05
4e+05
5e+05
50000
100000
150000
reference
combined_ref
gtdb_ref
scg_ref

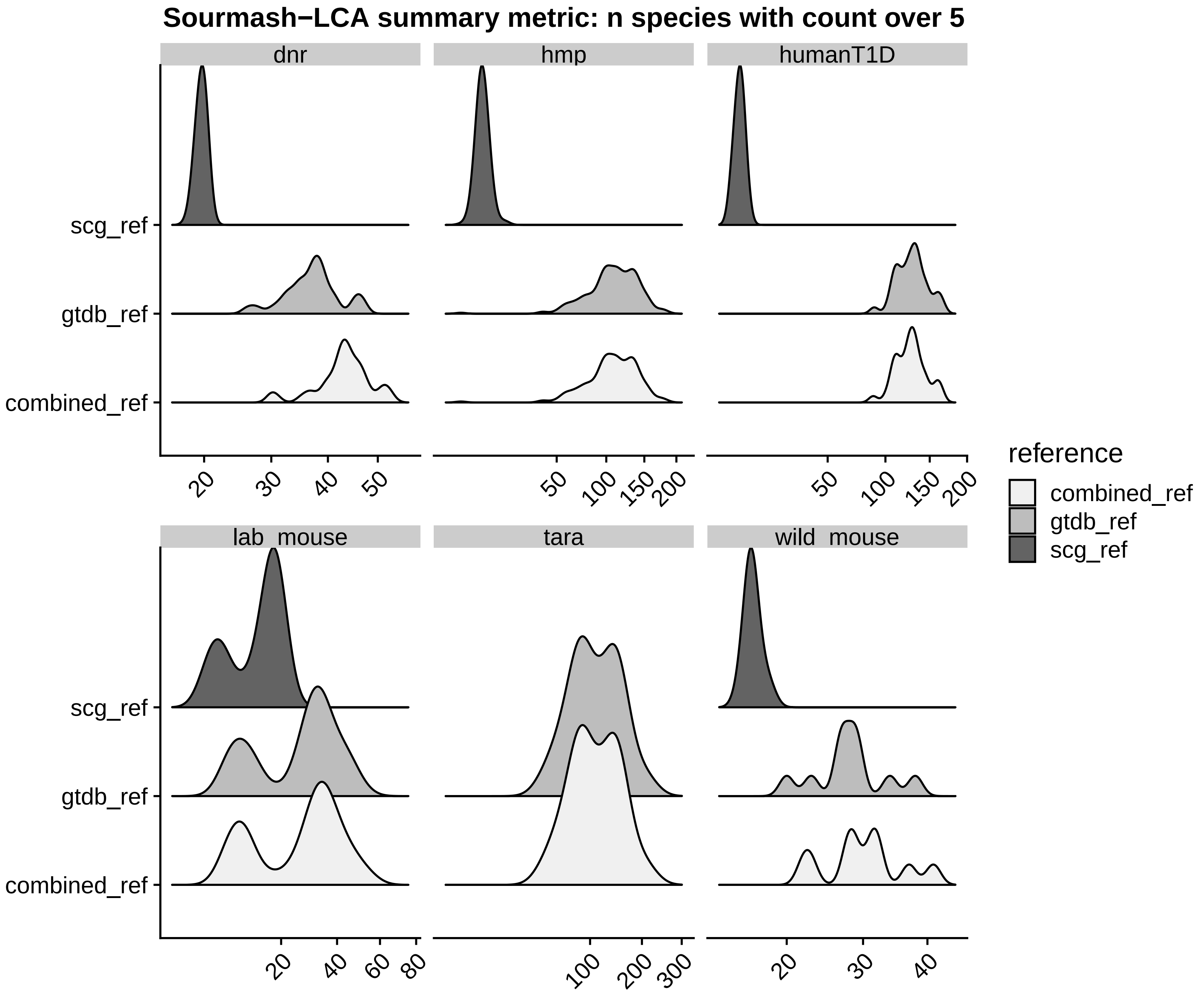
Sourmash−LCA summary metric: n species with count over 5
dnr
hmp
humanT1D
lab_mouse
tara
wild_mouse
scg_ref
gtdb_ref
combined_ref
20
30
40
50
50
100
150
200
50
100
150
200
20
40
60
80
100
200
300
20
30
40
reference
combined_ref
gtdb_ref
scg_ref
