# Supplementary material for: Single cell genome sequencing of laboratory mouse microbiota improves taxonomic and functional resolution of this model microbial community
Source: PLoS One. 2022 Apr 13;17(4):e0261795. doi: 10.1371/journal.pone.0261795 (PMC9007364; doi:10.1371/journal.pone.0261795)
Supplement: S3 Fig — Ridgeline plots representing distributions of 3 metagenomic classifier performance metrics when using MIDAS—mean coverage of 15 phylogenetically informative marker genes, median coverage of the same genes, and prevalence (number of samples a species is present in). Ridgeline plots are a form of multi-distribution density plot that vertically separate the individual distributions to improve clarity in cases of overlap, necessitating the removal of the traditional y axis label (“density”). This renders the densities not directly comparable between individual ridge lines, but aids in assessment of differences in skew, bimodality, and potential pronounced shift of distribution peaks. The plots are faceted by test metagenomic dataset, and each line within the facet reflects one of the three reference database options—default MIDAS v1.2 database (labeled “midas_db_v1.2”), a custom database with single-cell genomes only (labeled “midas_db_scg_only”), and a combined database with the MIDAS v1.2 and single cell genomes (labeled “midas_db_combined”). (PDF) [file pone.0261795.s005.pdf]

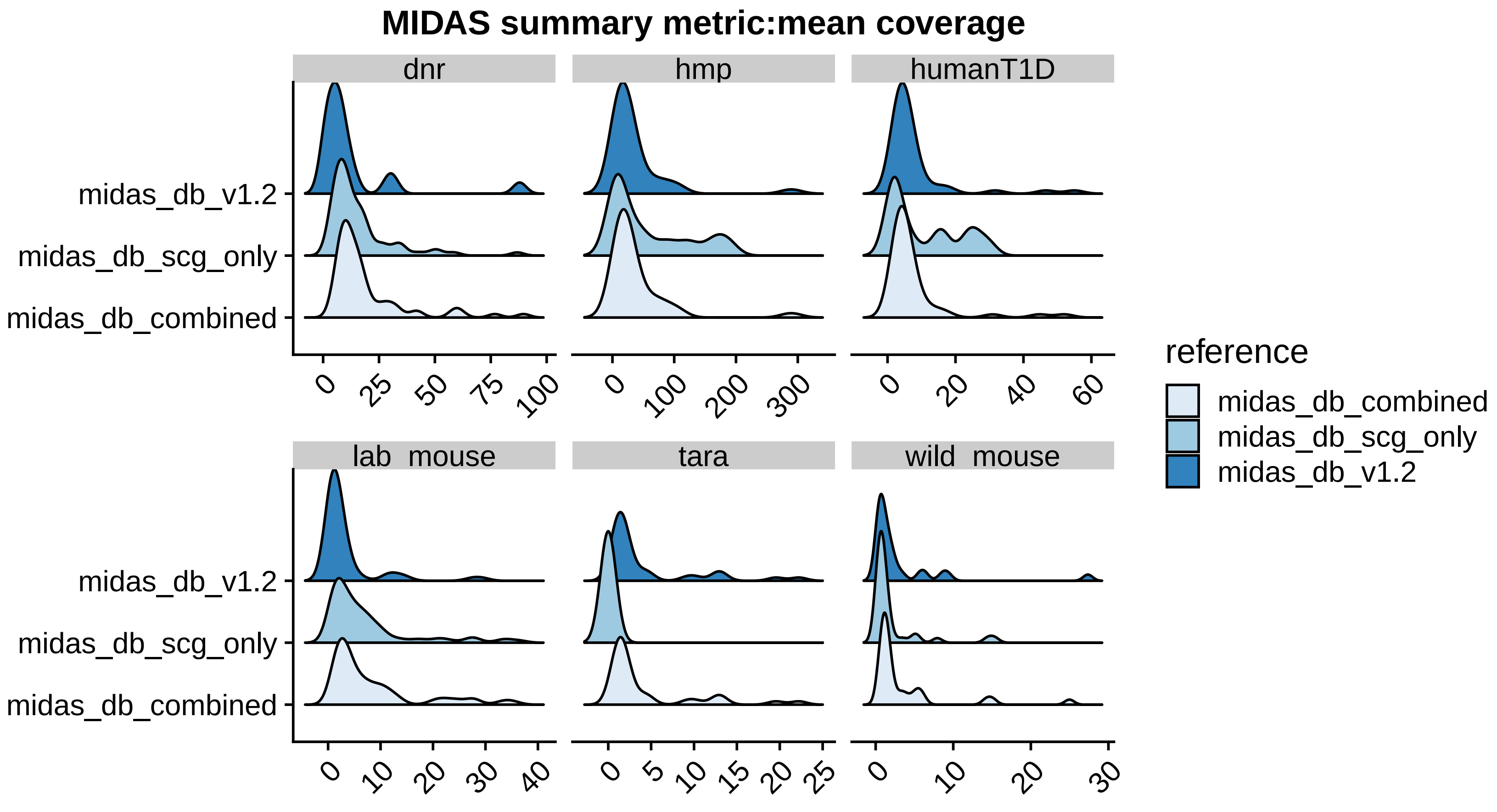
MIDAS summary metric:mean coverage
dnr
hmp
humanT1D
lab mouse
tara
wild mouse
midas_db_v1.2
midas_db_scg_only
midas_db_combined
reference
midas_db_combined
midas_db_scg_only
midas_db_v1.2

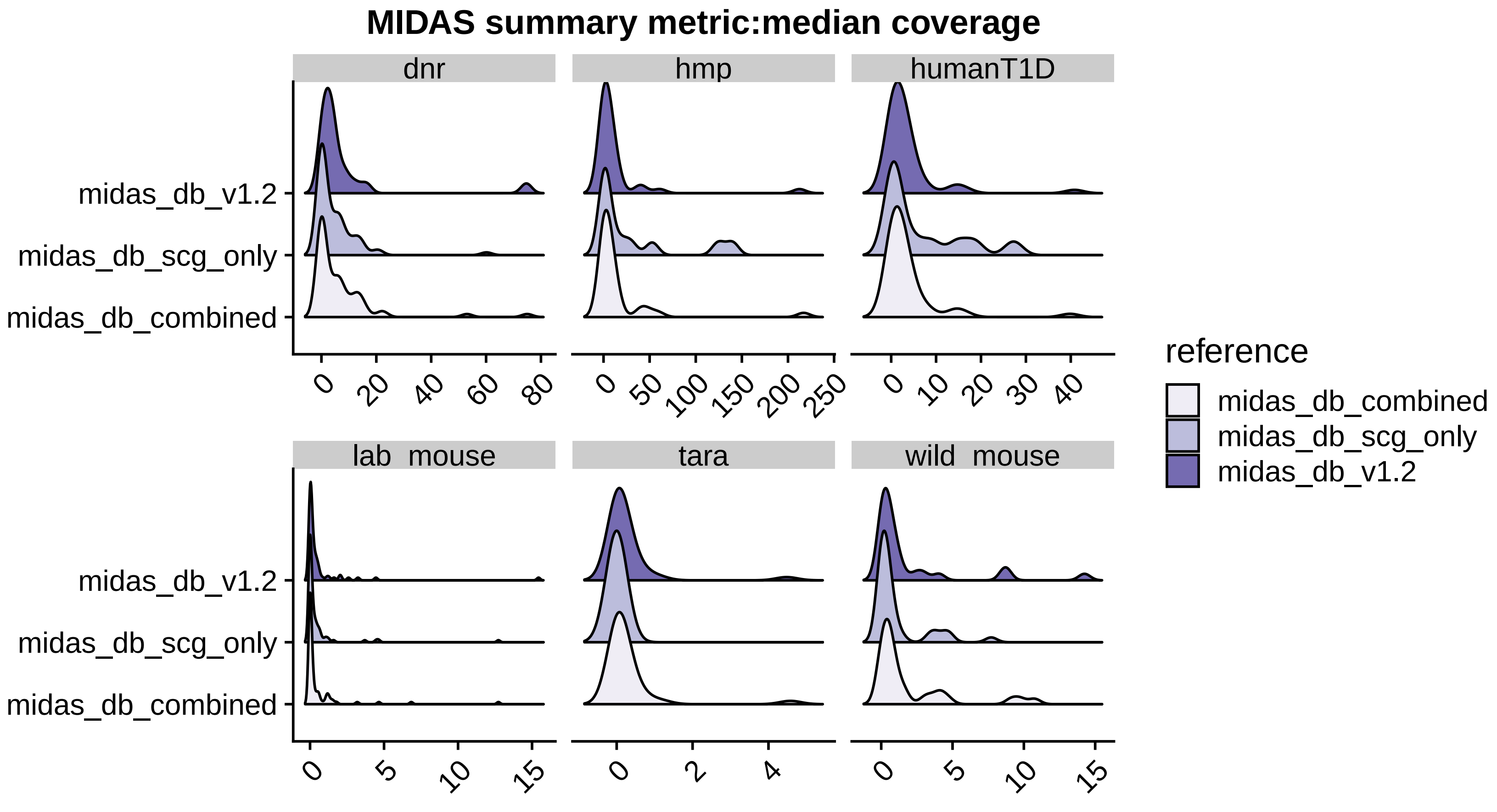
MIDAS summary metric:median coverage
dnr
hmp
humanT1D
lab mouse
tara
wild mouse
midas_db_v1.2
midas_db_scg_only
midas_db_combined
reference
midas_db_combined
midas_db_scg_only
midas_db_v1.2

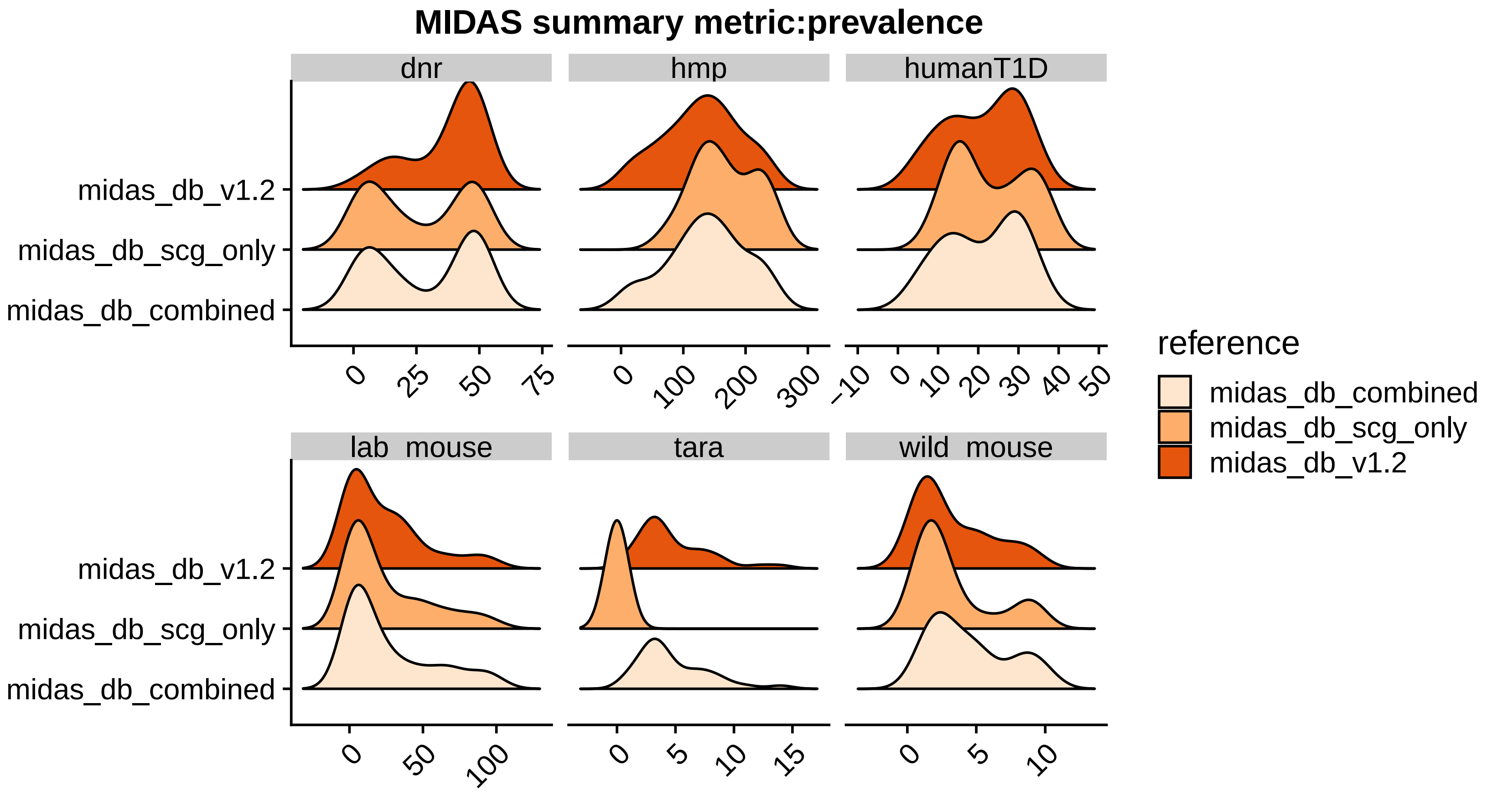
MIDAS summary metric:prevalence
dnr
hmp
humanT1D
lab mouse
tara
wild mouse
midas_db_v1.2
midas_db_scg_only
midas_db_combined
reference
midas_db_combined
midas_db_scg_only
midas_db_v1.2
